# Is PET–CT an accurate method for the differential diagnosis between chondroma and chondrosarcoma?

**DOI:** 10.1186/s40064-016-1782-8

**Published:** 2016-02-29

**Authors:** Reynaldo Jesus-Garcia, Akemi Osawa, Renee Zon Filippi, Dan Carai Maia Viola, Marcos Korukian, Guilherme de Carvalho Campos Neto, Jairo Wagner

**Affiliations:** Orthopedic Oncology Department, Hospital Israelita Albert Einstein, São Paulo, SP Brazil; Nuclear Medicine Department, Hospital Israelita Albert Einstein, São Paulo, SP Brazil; Surgical Pathology Department, Hospital Israelita Albert Einstein, São Paulo, SP Brazil

**Keywords:** Cartilage, Neoplasm, PET**–**CT, Bone tumors, Chondroma, Chondrosarcoma

## Abstract

The differential diagnosis between chondroma and intraosseous chondrosarcoma is based on imaging and clinical exams, but only a biopsy can confirm diagnosis. The aim of this study was to evaluate the value of PET–CT in differentially diagnosing chondroma and chondrosarcoma. From October 2009 to May 2015, 36 patients with cartilaginous bone lesions in the extremities, 12 (33.3 %) men and 24 (66.6 %) women, were prospectively included in the study. Patients ranged in age from 21 to 68 years, with a mean age of 44 years. Lesions were located in the long bones: in the proximal humerus in 26 (72.2 %) patients, in the femoral shaft in 1 (2.7 %), in the distal femur in 7 (19.4 %), and in the proximal tibia in 2 (5.5 %). The SUVmax value of 2.0 was used to separate between patients submitted to surgery and patients submitted to observation. Among the 36 patients studied, 17 (47.2 %) had SUVmax ≤ 2.0, and they were diagnosed as chondroma and they were treated conservatively. Follow-up ranged from 14 to 76 months, averaging 38 months. Nineteen (52.7 %) patients with SUVmax >2.0 were diagnosed as chondrosarcoma and underwent surgery. The area of the curve, calculated considering the SUV variable as numeric, is estimated in 0.966, with a 95 % confidence interval from 0.906 to 1.000. To evaluate the sensitivity, specificity and positive/negative predictive values, it was built a 2 × 2 table. Significance was set at p < 0.05. According the criteria of maximum sensitivity and specificity, the cut point suggested to SUVmax was 2.2. If we consider this point, it is possible to identify 19 of 36 positive cases to chondroma (52.8 %), it means, all chondrosarcomas of the series. We concluded that PET–CT can be used as an objective and quantitative method of differentiating between chondromas and chondrosarcomas located within the long bones. It represents a complementary examination to standard imaging (X-ray, scintigraphy, CT and MRI) and pathological exams. The SUVmax between 2.0 and 2.2 would be a range area between chondroma and chondrosarcoma and this range can be of value, among others exams, in decide the best treatment for patients with cartilaginous lesions in long bones.

*Level of evidence* Level I—diagnostic study—prospectively investigating a diagnostic test using a universally applied “gold” standard.

## Background

The differential diagnosis of intraosseous cartilaginous lesions is based on imaging or clinical examination findings. Chondroma is a benign variant, characterized by the formation of mature hyaline cartilage without atypia, while chondrosarcoma is a malignant tumor that produces atypical cartilage matrix and features an infiltrative growth pattern in the medullary and cortical bone tissue.

The differential diagnosis between benign and malignant variants based on imaging is not reliable and often results in false negatives or false positives (Rosenthal et al. 1984).

One option to avoid false results is to conduct a biopsy of the tumor for pathological analysis. However, samples collected from a single area of the lesion are not representative of the entire lesion, since the tumor may have niches of malignant transformation into chondrosarcoma next to areas of morphologically benign cartilage.

Another issue to consider when doing a biopsy is that histologically differentiating between chondromas and low-grade chondrosarcomas may be difficult even for experienced pathologists, mainly because differentiation is observer-dependent, especially when only a small tissue sample is available (Evans et al. 1977; Mankin et al. 2006).

Image-guided biopsies in patients with cartilage lesions have a low accuracy of 85.9 % (Jennings et al. 2010). When we compare the diagnosis based on the biopsy and the histological final grade after studying the whole tumor, we usually find a high rate of discrepancies (Kumar et al. 1993; Olszewski et al. 1983).

Studies on the use of PET**–**CT in the study of sarcomas began to appear after 2001 (Al-Ibraheem et al. 2013; Aoki et al. 2001, 2003; Benz et al. 2009, 2010; Brenner et al. 2004; Eary et al. 1998, 2002; Feldman et al. 2005; Folpe et al. 2000; Garcia et al. 1996; Purandare et al. 2009; Schulte et al. 1999). Some authors suggested that because PET**–**CT detects hyper metabolic foci, that whole body PET**–**CT could be an important test for identifying chondrosarcomas and their recurrence after surgery. Charest et al. (2009) retrospectively evaluated the sensitivity of PET**–**CT in the diagnosis of sarcomas of bone and soft tissue and established a SUVmax score of 2.5 as the threshold between low and high-grade sarcomas, with an accuracy of 94 %.

In 2005, Feldman et al. (2005) studied the applications of PET**–**CT in the differential diagnosis between 29 benign and malignant cartilaginous lesions and used a “cutoff” of SUV = 2.0. The SUVmax was >3.3 in grade I chondrosarcomas, >5.4 in grade II and >7.1 in grade III chondrosarcomas. They found the method to have high sensitivity, specificity and accuracy. They concluded that an SUV value >2.0 was a suspected malignancy (with 91 % sensitivity; Feldman et al. 2005).

Benz et al. (2009, 2010) studied the accuracy and sensitivity of PET**–**CT in evaluating the response of sarcomas to neoadjuvant treatment and found that the best responders showed greater declines in SUV levels relative to poor responders. These authors concluded that PET**–**CT could accurately detect lymph nodes and metastases in patients with sarcomas (Benz et al. 2009, 2010)

Despite previous publications evaluating PET**–**CT in chondrosarcomas and in sarcomas in general, we found no study evaluating PET**–**CT in the differential diagnosis between chondromas and chondrosarcomas with the aim of determining whether tumor removal should be indicated.

We conducted a prospective study comparing the sensitivity and accuracy as well as the false positive and false negative rates of PET**–**CT in patients with cartilaginous tumors in the appendicular skeleton.

The aim of this study was to evaluate the validity of PET**–**CT as a method for the differential diagnosis between chondroma and chondrosarcoma in patients with cartilaginous neoplasms of long bones.

## Results

Among all 36 patients, 17 (47.2 %) with SUVmax scores ≤2.0 were submitted to PET**–**CT and diagnosed, by the PET**–**CT interpretation, as chondroma. These patients were not submitted to surgery. Nineteen (52.8 %) patients had a SUVmax > 2.0 and were diagnosed, by the PET**–**CT interpretation, as chondrosarcoma. These patients were submitted to surgery.

The result of the pathological examination showed that among the 19 (52.8 %) patients, with SUVmax > 2.0, 18 (50.0 %) were confirmed as chondrosarcoma grade I and 1 (2.7 %) patient did not confirm the PET**–**CT results and had the final diagnosis as chondroma.

At the last follow-up, no patients among the 17, not submitted to surgery, had evidence of lesion progression, which supported the diagnosis of chondroma.

When we analyzed the pathologic results in light of the PET**–**CT findings, we found 1 chondrosarcoma among the 17 patients with SUVmax ≤ 2.0, (Patient # 29, SUVmax = 2.0). On the other hand, we found 18 (50.0 %) chondrosarcomas and 1 (2.7 %) chondroma on the pathology analysis, among the 19 patients with SUVmax > 2.0 (Table 1).

**Table 1:** SUVmax in patients with and without surgical indication relative to results obtained in the last follow-up

SUVmax	Positive (chondrosarcoma)	Negative (chondroma)	Total
Positive (>2.0)	18	1	19
Negative (≤2.0)	1	16	17
Total	19	17	36

Golden standard (anatomopathological report or last follow-up)

During follow-up of at least 14 months (14–76 months, medium = 40 months), to patients with chondroma diagnosis, we observed no cases of progression of the lesion, based on MRI and clinical evaluation.

The accuracy of the SUVmax in the differentiation between chondroma and chondrosarcoma was evaluated, concerning the numeric value, by a ROC curve. The statistical analysis, concerning the categorical variable, was evaluated among the true positives, true negatives, false positives and false negatives values. The measure of accuracy, sensitivity, specificity, prevalence, and the predictive positive and negative were evaluated and followed in the confidence range of 95 %.

The diagnosis based on anatomopathological examination or in the follow up presented 19 (52.8 %) among 36 cases as chondrosarcoma. One of the patients with diagnosis of chondroma, was submitted to surgery. When we consider the value of SUVmax bigger than 2.0, it was possible to identify 18 among 36 cases positive to chondrosarcoma (63.9 %). One patient had the diagnosis of chondroma.

The area of the curve, calculated considering the SUV variable as numeric, is estimated in 0.966, with a 95 % confidence interval from 0.906 to 1.000. To evaluate the sensitivity, specificity and positive/negative predictive values, we built a 2 × 2 table. Kappa agreement was computed using the SPSS statistical package and significance was set at p < 0.05. According the criteria of maximum sensitivity and specificity, the cut point suggested to SUVmax was 2.2. If we consider this point, it is possible to identify 19 of 36 positive cases to chondroma (52.8 %), it means, all chondrosarcomas of the series.

The measures of diagnostic adequacy were calculated considering the two points of cut. They were presented in the Table 2. They indicate the method as more sensible than specific. But, the range of the confidence interval indicate that more patients will be necessary to evaluate the SUVmax as a tool to discriminate the chondroma versus chondrosarcomas patients (Landis and Koch 1977).

**Table 2:** Adequacy diagnostic measures

	SUV ≥ 2.0 Estimate (IC 95 %)	SUV ≥ 2.2 Estimate (IC 95 %)
True positives	19	18
False positives	4	1
False negatives	0	1
True negatives	13	16
Prevalence by SUV	63.9 % (46.2–79.2 %)	52.8 % (35.5–69.6 %)
Real Prevalence	52.8 % (35.5–69.6 %)	52.8 % (35.5–69.6 %)
Sensitivity	100.0 % (75.1–100.0 %)	94.7 % (74.0–99.9 %)
Specificity	76.5 % (50.1–93.2 %)	94.1 % (71.3–99.9 %)
Positive predictive value	82.6 % (61.2–95.0 %)	94.7 % (74.0–99.9 %)
Negative predictive value	100.0 % (66.1–100.0 %)	94.1 % (71.3–99.9 %)
Accuracy	88.9 % (73.9–96.9 %)	94.4 % (81.3–99.3 %)

## Discussion

The differential diagnosis between chondroma and chondrosarcoma remains one of the toughest in Orthopedics Oncology. We prospectively studied the validity of PET**–**CT for this purpose.

When we analyze the differences between chondroma and chondrosarcoma we found non specific symptoms and the clinical and orthopedic examination are often normal or uncharacteristic.

The X-ray and CT scan provides good definition images of cartilage, but sometimes, are unable to differentiate between benign and malignant cartilage. X-ray and CT are very useful for the analysis of cortical bone invasion and periosteal reaction by the tumor. If there is invasion, it is most likely that the lesion is a chondrosarcoma and not a chondroma, but most of the times, the findings unfortunately are inconclusive.

Skeletal scintigraphy with technetium reveals a slight increase in concentration in chondromas. The concentration is greater in chondrosarcoma lesions, particularly in the more peripheral areas of an active lesion, which cause erosion and cortical bone reaction. However, because these are slow-growing lesions, even in the case of chondrosarcomas, bone destruction, which causes neogenesis, is small.

In MRI, chondromas present with low or medium signal on T1-weighted sequences and high signal on T2 sequences. The erosion and remodeling of cortical bone and extra-cortical involvement appear clearly. Injection of gadolinium increases the signal, but sometimes, even with contrast, it is difficult to differentiate between chondroma and chondrosarcoma (Aoki et al. 1991; Som et al. 1980).

These methods are not a 100 % precise, and interpretation can vary among expert radiologists (Skeletal Lesions Interobserver Correlation among Expert Diagnosticians (SLICED) Study Group 2007). Often, the radiologist emits an inconclusive report of “compatible with chondroma or chondrosarcoma” or a report of “cartilaginous lesion”.

For the definitive anatomopathological diagnosis differentiating chondroma and chondrosarcoma, all available information must be considered (patient age, presence of pain, history of rapid growth, lesion location, size of the lesion, radiographic, CT, magnetic resonance and scintigraphy image). However, the cartilaginous tumor biopsy is controversial and currently most bone tumor reference centers do not perform it. The cartilaginous tumor is heterogeneous in its presentation and biopsy sampling of a region may not represent a significant area of the tumor.

Histological staging is the most important topic regarding the evolution of the biological behavior of chondrosarcoma and is mainly based on cellularity and atypia or the presence of bizarre morphology of the cells (Aoki et al. 1991; Brien et al. 1997; Lee et al. 1999; Sundaram and McLeod 1990).

We understand that a weak point of this analysis is to define the golden standard as the anatomopathological report. We know that the reliability of the grading of cartilaginous neoplasm, even among specialized and experienced pathologists is critical, but our analysis, is based on Evans criteria, as a protocol in our Institution, and we believe there is no important bias in the interpretation (Evans et al. 1977; Skeletal Lesions Interobserver Correlation among Expert Diagnosticians (SLICED) Study Group 2007).

Several signaling pathways have been shown to be affected in central cartilaginous neoplasms: RB1 and TP53, cytogenetic alterations and mutations in the IDH1 and IDH2 genes, as well as the analysis of DNA ploidy by cytofluorometry, which has improved the knowledge of the origin and real nature of this type of lesion. However, these findings do not yet translate into useful diagnostic tools for the differential diagnosis between chondroma and chondrosarcoma, which would be possible only after tumor resection (Aoki et al. 2003; Brien et al. 1997; Brien et al. 1999).

Under these circumstances, and considering the doubts raised by the imaging findings, we decided to complement the workup with the use of PET**–**CT.

PET**–**CT has the ability to measure the avidity of malignant cells by glucose, since the intracellular transport of glucose labeled with 18-FDG ([18F] Fluoro-2-deoxy-d-glucose) is higher in malignant cells. The low permeability of the membrane limits the back-diffusion of FDG during the examination, keeping the FDG within the malignant cells, which allows for their detection (Schulte et al. 1999).

Based on evidence in the literature showing that it is possible to differentiate benign lesions with low SUVmax from malignant lesions with high SUVmax, we conducted a prospective evaluation of intraosseous cartilage lesions of long bones (Dehdashti et al. 1996; Eary et al. 2011; Kern et al. 1988; Schulte et al. 2000). We limited our evaluation to intra osseous lesions in humerus, femur and tibia. We defined the value of the uptake of radioactive fluorine-labeled glucose as SUVmax = 2.0 for the dividing line between patients whose will be submitted to surgical treatment supposed to be chondrosarcomas and patients without surgical treatment, supposed to be chondromas (Feldman et al. 2005).

Unique properties of cartilage (e.g., small cellularity, very low rate of mitosis, high quantities of chondroid matrix and inactive extracellular matrix, poor vascularization and anaerobic glycolysis) contribute to low SUV values. Even in vascularized or aggressive cartilaginous lesions, SUV levels rarely reach the levels of sarcomas with a different histogenesis, such as osteosarcomas, fibrosarcomas or Ewing’s tumor (Brenner et al. 2004; Schulte et al. 2000). Because of this, grade I and II chondrosarcomas, regardless of size, amount of calcification or necrosis areas, have a low SUV, reflecting glucose metabolism.

We agree with Brenner et al. (2004) that the metabolism of the tumor, in terms of metabolic activity and oncologic behavior, is characterized by SUVmax. The area with the highest SUV reflects the area in the tumor with the highest metabolic activity (which represents the most aggressive area of the lesion), and it is this area that should be used for tumor classification, treatment decisions and prognosis. This area is the site of greatest activity in the lesion, regardless of tumor size or shape. Moreover, SUVmax allows for a better comparison between different devices and services, as it does not depend on the definition of the volume or shape of the ROI, which is examiner-dependent. We believe that the SUVmax, obtained manually inside the ROI placed over the tumor, is the measure of greatest reproducibility.

The literature shows that SUVmax values for the differentiation between benign and malignant tumors vary between 1.3 and 4.0 across Institutions and publications, due to the different equipment and protocols used, as well as differences in lesion histology (Eary and Conrad 2011; Eary et al. 2002).

Although we used a value of SUVmax in our study, we believe that the dividing line between chondroma and chondrosarcoma should be a range of SUVmax scores and not a cutoff line, with a specific SUVmax value. If we have used the SUVmax ≥ 2.2 it would be possible to detect 18 among the 19 cases of chondrosarcoma and 16 among the 17 cases of chondroma. It was demonstrated in the Table 2. The best option would be considering the range between SUVmax = 2.0 and 2.2 as an intermediate area when we use the PET**–**CT to differentiate the chondroma from the chondrosarcoma.

Some variables can alter the assessment of cartilage lesion SUV, such as small size, the time between injection and the start of the test, the duration of the test post-injection, the amount of glucose in the patient’s blood, patient weight and body surface area. The small number of patients and the short follow-up time of patients not undergoing surgery may represent a weak point in our conclusions, but we believe that our results nevertheless open a new perspective on the noninvasive diagnosis of cartilaginous tumors.

We also believe that creating a score that incorporates data from the clinical examination as well as X-ray, CT, MRI, scintigraphy and PET**–**CT could increase diagnostic accuracy.

Treatment for chondromas is different from that for chondrosarcomas. Benign cartilaginous lesions can be treated conservatively. When we face a chondrosarcoma, the curettage plus cryotherapy and cementation or wide resection would be the most frequent options of treatment. We have to consider the possibility of the progression of the cancer cells inside the medullary canal and destruction of the cortical bone reaching the soft tissue, with a chance of progressing to the extra-cortical compartment and invading the soft tissue as well as metastasizing to the lungs. Early diagnosis of chondrosarcoma is important in oncological practice.

One point that may be questioned in our study is the fact that we did not perform histopathology on benign tumors, those who had the SUVmax ≤ 2.0, and only conducted follow-up for at least 14 months. Follow-up without a anatomopathological exam may lead to false negatives, which can only be clarified with a longer follow-up. However, biopsies of cartilage lesions also would lead to a high number of false negatives, which could also represent a fragile and ethically questionable point in a study such as this one. In addition to the risk of implantation of cartilaginous cells during the biopsy, there are risks associated with anesthesia, infection and the hospital costs for the biopsy and anatomopathological examination.

PET**–**CT is not without risks, as it involves the injection of contrast as well as some radiation from the tomography. Thus, we suggest that, in the future, the CT scan should be limited to the area with bone changes in order to limit the amount of radiation to the site being studied. However, this would not allow us to conduct a comprehensive staging of the patient, including a CT scan of the chest, which is important in the case of chondrosarcomas. PET**–**CT is a costly procedure that is not yet available in all Hospitals, but has the potential to soon become an important tool in the differentiation between chondroma and chondrosarcoma.

The confidence interval of this study was relatively large (which is most likely due to the small sample size). Further studies with a more robust sample size are needed.

We believe that a larger number of patients will allow us to confirm or not, if the use PET**–**CT, to differentiate between patients with cartilaginous lesions, who require surgical treatment from those who do not is a method of value.

## Conclusions

PET**–**CT can be used as an objective and quantitative method of differentiating between chondromas and chondrosarcomas located within the long bones. It represents a complementary examination to standard imaging (X-Ray, scintigraphy, CT and MRI) and pathological exams. The SUVmax between 2.0 and 2.2 would be a range area between chondroma and chondrosarcoma and can be of value, among others exams, in decide the best treatment for patients with cartilaginous lesions in long bones.

## Methods

The study was submitted and approved by the Ethics Committee of the Institution and all patients gave their writing consent to participate in this prospective study.

From October 2009 to May 2015, 36 patients with cartilage lesions detected through imaging were staged using X-rays, CT, MRI and PET**–**CT (Table 3). Twenty-four (66.6 %) patients were female and 12 (33.3 %) were male, with a mean age of 44.0 (range 21–68). Lesions were located in the long bones: in the proximal humerus in 26 (72.2 %) patients, in the femoral shaft in 1 (2.7 %), in the distal femur in 7 (19.4 %), and in the proximal tibia in 2 (5.5 %). All patients were submitted to X-rays, CT, scintigraphy, and an MRI and were then sent for PET**–**CT scan.

**Table 3:** Patients, demographics and results

Order	Sex	Age	Site	Size (MRI) in millimetres	SUVmax	Date of PET (day/month/year)	Follow-up (months)	Treatment	Date of surgery (day/month/year)	Synthesis	Last follow-up (day/month/year)	Final diagnosis
1	Female	65	Femur diaphysis	140 × 25 × 23	1.90	28-10-09	61	Conservative	Void	No	23-10-14	Enchondroma
2	Male	48	Distal femur	65 × 21 × 19	1.80	05-08-10	58	Conservative	Void	No	08-05-15	Enchondroma
3	Female	34	Proximal humerus	20 × 18 × 8	3.40	23-09-10	55	Curetage + cement	22-09-2010	No	30-03-15	Chondrosarcoma1
4	Female	31	Proximal humerus	40 × 30 × 17	4.30	03-03-11	48	Curetage + cement	23-03-2011	No	23-02-15	Chondrosarcoma1
5	Female	48	Proximal humerus	40 × 30 × 20	1.40	25-05-11	48	Conservative	Void	No	25-04-15	Enchondroma
6	Female	42	Proximal humerus	44 × 21 × 20	1.60	19-07-11	46	Conservative	Void	No	14-04-15	Enchondroma
7	Female	56	Proximal humerus	66 × 25 × 19	1.70	12-09-11	50	Conservative	Void	No	01-11-15	Enchondroma
8	Male	58	Proximal humerus	32 × 22 × 28	2.00	15-10-11	51	Conservative	Void	No	10-12-15	Enchondroma
9	Female	61	Proximal humerus	38 × 25 × 22	2.00	19-01-12	46	Conservative	Void	No	23-10-15	Enchondroma
10	Female	58	Proximal humerus	38 × 22 × 18	1.90	20-01-12	45	Conservative	Void	No	03-10-15	Enchondroma
11	Male	41	Distal femur	70 × 28 × 20	2.80	02-05-12	37	Curetage + cement	11-06-2012	Plate and screws	03-05-15	Chondrosarcoma1
12	Female	31	Proximal humerus	35 × 30 × 27	1.50	28-08-12	32	Conservative	Void	No	31-03-15	Enchondroma
13	Female	45	Distal femur	33 × 26 × 24	2.60	31-08-12	31	Curetage + cement	02-10-2012	No	01-04-15	Chondrosarcoma1
14	Male	34	Proximal humerus	38 × 22 × 20	5.80	26-10-12	39	Curetage + cement	12-12-2012	No	19-01-16	Chondrosarcoma1
15	Female	36	Distal femur	41 × 18 × 16	4.20	03-11-12	29	Curetage + cement	12-12-2012	No	31-03-15	Chondrosarcoma1
16	Female	54	Proximal Tibia	21 × 14 × 14	2.00	15-12-12	38	Conservative	Void	No	20-01-16	Enchondroma
17	Female	46	Distal femur	29 × 20 × 19	3.20	17-01-13	28	Curetage + cement	27-02-2013	Plate and screws	28-04-15	Chondrosarcoma1
18	Male	30	Proximal humerus	56 × 18 × 17	4.30	18-03-13	25	Curetage + cement	05-06-2013	No	25-03-15	Chondrosarcoma1
19	Female	43	Proximal humerus	40 × 25 × 20	2.30	09-04-13	25	Curetage + cement	24-04-2013	No	20-04-15	Chondrosarcoma1
20	Female	31	Proximal humerus	150 × 30 × 28	2.90	27-07-13	20	Curetage + cement	15-08-2013	No	30-03-15	Enchondroma
21	Female	68	Proximal humerus	58 × 32 × 30	2.90	15-08-13	20	Curetage + cement	28-08-2013	No	20-04-15	Chondrosarcoma1
22	Female	43	Distal femur	36 × 15 × 17	2.80	16-10-13	26	Curetage + cement	30-10-2013	No	17-12-15	Chondrosarcoma1
23	Male	60	Proximal humerus	15 × 18 × 16	1.95	03-02-14	22	Conservative	Void	No	25-11-15	Enchondroma
24	Female	38	Proximal humerus	70 × 25 × 20	0.30	03-09-09	76	Conservative	Void	No	20-11-15	Enchondroma
25	Female	42	Proximal humerus	24 × 16 × 20	1.70	28-11-13	25	Conservative	Void	No	20-12-15	Enchondroma
26	Female	42	Proximal humerus	85 × 25 × 25	3.09	25-03-14	21	Curetage + allograft	20-06-2014	Plate and screws	21-12-15	Chondrosarcoma1
27	Male	21	Proximal humerus	51 × 18 × 16	2.50	15-05-13	33	Curetage + cement	15-07-2014	No	26-01-16	Chondrosarcoma1
28	Male	53	Proximal humerus	29 × 24 × 20	1.72	26-08-14	17	Conservative	Void	No	25-01-16	Enchondroma
29	Female	29	Distal femur	31 × 22 × 15	2.00	05-11-14	12	Curetage + cement	26-11-14	No	01-11-15	Chondrosarcoma1
30	Male	39	Proximal Tibia	57 × 13 × 24	2.20	15-12-14	9	Curetage + cement	28-08-15	Plate and screws	08-09-15	Chondrosarcoma2
31	Male	46	Proximal humerus	84 × 21 × 10	0.00	27-11-14	14	Conservative	Void	No	19-01-16	Enchondroma
32	Female	31	Proximal humerus	32 × 30 × 20	0.00	12-11-14	14	Conservative	Void	No	18-01-16	Enchondroma
33	Female	39	Proximal humerus	54 × 29 × 26	3.20	27-02-15	10	Curetage + cement	25-03-15	No	15-12-15	Chondrosarcoma1
34	Male	49	Proximal humerus	20 × 17 × 19	2.70	05-05-15	8	Curetage + cement	05-05-2015	Kirschner wires	29-12-15	Chondrosarcoma1
35	Female	50	Proximal humerus	15 × 15 × 10	2.37	29-05-15	8	Curetage + cement	03-06-2015	No	19-01-16	Chondrosarcoma1
36	Male	33	Proximal humerus	76 × 17 × 15	3.30	16-11-15	2	Curetage + cement	25-11-2015	Plate and screws	27-01-16	Chondrosarcoma1

PET**–**CT was performed in all patients during the staging period. Patients underwent a preparation with a low-carbohydrate diet for 12 h before the test and fasted for 4 h (but could drink water). Patients had their blood glucose measured before injection of the radioactive tracer and all results were below 180 mg/dL. Sixty to ninety minutes before the start of the study, they received an intravenous injection of 0.1 mCi/kg (3.7 MBq/kg) 18F-FDG and remained at rest in a quiet environment during the period of accumulation.

The images were obtained with hybrid PET**–**CT equipment. Until May of 2012, tests were performed with Discovery ST (General Electric—USA) equipment, with 3D acquisition, 4 min per FOV and reconstruction with Ultra HD-PET with 2 iterations and 21 “subsets”. After May of 2012, scans were performed with the Biograph mCT 40 PET**–**CT machine (Siemens Medical Solutions, USA), 3D acquisition, 3 min per FOV and Ultra HD-PET reconstruction with 2 iterations and 21 subsets. On both equipment, CT was conducted before PET, scanning from the skull to the distal femur (extending to the whole body in cases of lesions below the knee) with low doses of radiation and applying attenuation correction. On the Discovery ST-GE equipment, the irradiation dose used was 120 kV and on the Biograph mCT 40 Siemens equipment, it was 100 kV. In both equipment, radiation beam modulation was applied.

**Fig. 1:**
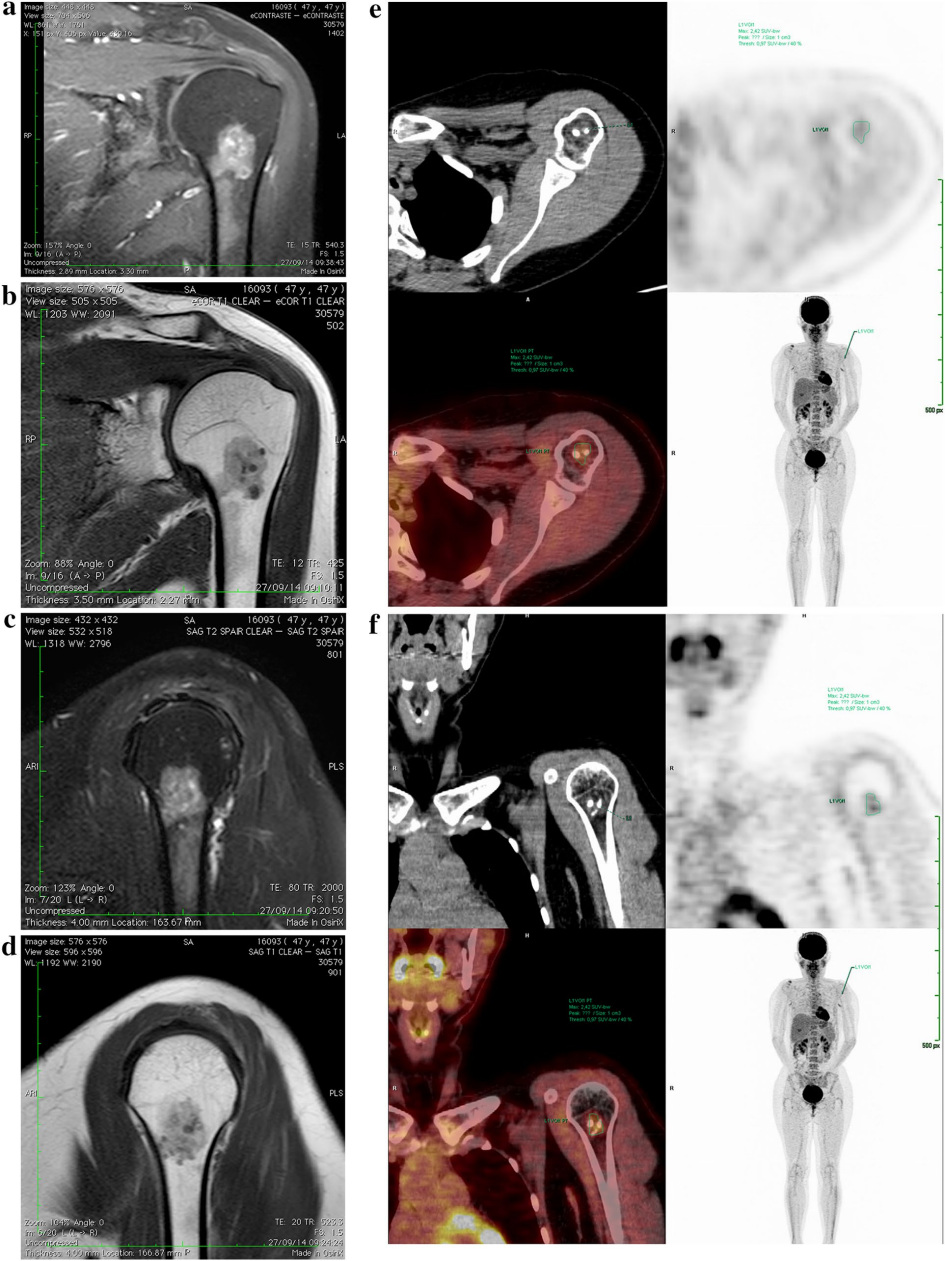
Example of an MRI image of the shoulder, where it is not possible to confirm whether the lesion is a chondroma or a chondrosarcoma. In **a** axial T2 MRI image of the proximal humerus, and **b** T1 image. In **c** and **d**, the bone lesion can be visualized in T2 and T1 images. In **e**, axial PET and in **f** coronal images show the proximal region of the left humerus. Note the PET**–**CT presenting the volumetric region of interest (VOI) with an SUVmax = 2.0

All tests were processed at the same workstation, the Syngo.via (Siemens Medical Solutions, USA), with PET**–**CT software. We performed an automatic volumetric region of interest (VOI) in the chondral lesion in order to obtain the automatic SUV value (40 % threshold) of the region of interest. The analysis was redone in some situations, for instance, when the automatic VOI included degenerative changes with increased glycolytic activity (when the lesion was close to joints), shifting the area of interest. We thus avoided including this region in the analysis (Fig. 1).

Among the 36 study patients, 17 (47.2 %) had SUVmax ≤ 2.0. Follow-up ranged from 14 to 76 months, with a mean of 38 months. Sixteen of these patients were treated conservatively (without surgery) and their follow-up was done only with MRI every 6 months during the first 2 years and once a year from the third to the fifth years. No patient among them presented tumor progression or metastasis during the follow-up period. One patient (Pat.#29), with SUVmax = 2.0, but with an MRI reported as chondrosarcoma was submitted to surgery.

The 19 (52.7 %) patients with SUVmax > 2.0 underwent surgery. The tumor removed during surgery was sent for pathological examination. The material was fixed in a 10 % formalin solution, decalcified with 15 % nitric acid, subjected to routine histology and stained with hematoxylin and eosin. According to the current literature, the criteria used for histologic diagnosis are based on lesion cellularity, the presence of permeation of the cortical and/or cancellous bone tissue, cytological atypia and the presence of myxoid degeneration in the matrix (Evans et al. 1977).

 The follow-up of the patients submitted to surgery was done with X-rays and MRI every 6 months during the first 2 years and once a year from the third to the fifth years. No patient present local recurrence or metastasis in the follow-up period.
